# Metabolic complementation between cells drives the evolution of tissues and organs

**DOI:** 10.1098/rsbl.2024.0490

**Published:** 2024-11-20

**Authors:** Mihaela Pavlicev, J. DiFrisco, Alan C. Love, Günter P. Wagner

**Affiliations:** ^1^ Department of Evolutionary Biology, University of Vienna, Vienna, Austria; ^2^ Complexity Science Hub, Vienna, Austria; ^3^ Konrad Lorenz Institute of Evolution and Cognition Research, Klosterneuburg, Austria; ^4^ Theoretical Biology Lab, The Francis Crick Institute, London, UK; ^5^ Department of Philosophy & Minnesota Center for Philosophy of Science, University of Minnesota, Minneapolis, MN, USA; ^6^ Yale University, New Haven, CT, USA; ^7^ Texas A&M, Hagler Institute for Advanced Study, College Station, TX, USA

**Keywords:** self-maintenance, levels of organization, tissue origin, metabolic complementation, metabolic constraint

## Abstract

Although evolutionary transitions of individuality have been extensively theorized, little attention has been paid to the origin of levels of organization within organisms. How and why do specialized cells become organized into specialized tissues or organs? What spurs a transition in organizational level in cases where the function is already present in constituent cell types? We propose a hypothesis for this kind of evolutionary transition based on two features of cellular metabolism: metabolic constraints on functional performance and the capacity for metabolic complementation between parenchymal and supporting cells. These features suggest a scenario whereby pre-existing specialized cell types are integrated into tissues when changes to the internal or external environment favour offloading metabolic burdens from a primary specialized cell type onto supporting cells. We illustrate this process of ‘supra-functionalization’ using the nervous system and pancreas.

## 1. A different kind of evolutionary transition

A central aim of evolutionary biology is to understand the origin and diversification of traits. Traditionally, it is assumed that a new trait is associated with a novel function [1,2]. Examples include implantation in mammals [3], hypsodonty in ruminants [4] and pharyngeal jaws of cichlids [5]. However, many organs perform functions that existed *prior* to the origin of the organ. For example, cells that produce digestive and detoxifying enzymes in early branching chordates pre-date the origins of the liver and pancreas [6–8], neural cells pre-date the central nervous system (CNS) and specific endocrine cells pre-date endocrine organs [9]. These observations imply that the origins of some novelties are not associated with the origin of the function they perform. Instead, organs and tissues can arise by the integration of functionally specialized cell types into a higher level unit. This raises new questions: how and why do specialized cells become organized into a tissue or an organ? What spurs a transition from cells to tissues when the function is already present?

Recently, there has been a proliferation of work on animal tissues [10–12] that leverages new experimental tools (e.g. single-cell transcriptomics). Since distributed cells perform the same function in some extant organisms that is performed by tissues or organs in others, empirical questions about the transition from one mode to another can be pursued.

Here we propose a hypothesis for tissue and organ origination that is motivated by the above observations. Our hypothesis is based on the recognition that cells have limited metabolic capacity but can also complement each other’s metabolic needs. This creates the potential for cells to integrate into a higher level unit through complementation, which ultimately leads to *interdependence* among the cells and thus to the evolutionary stability of a new level of organization—a tissue. We present preliminary evidence for this scenario of an ‘organizational transition’, highlight several predictions and discuss the hypothesis in the context of ideas about major evolutionary transitions.

## 2. Tissues and organs

Organization in a biological system is any configuration of entities that, through its internal structure and interactions, takes on unit integrity. Tissues are the epitome of organization because they integrate cell types into a coherent, functional unit. A tissue is a configuration of cell types and the extracellular matrix (ECM). Most animal tissues include a parenchymal cell type, which is dedicated to the function distinctive of the tissue, as well as tissue-specific fibroblasts and macrophages, endothelial cells and a variable cast of immune and ancillary cells. The parenchymal cells are often epithelial cells, which tend to be fixed in place in sheets, and which require nutrient support to pass through the basal membrane. In these tissues the supporting roles are played by mesenchymal cells, which can often move more freely. In mesodermal tissues (e.g. muscle, bone), the parenchymal cells may be mesenchymal, whereas in certain ectodermal tissues (e.g. cochlea), both parenchymal and supporting cells are epithelial. The hybrid epithelial–mesenchymal nature of many tissues and organs is reflected in the fact that they largely develop from epithelial–mesenchymal interactions [13]. Exceptions are the chordate CNS, which arises from an epithelial structure, the neural tube, and cartilage, which consists of a single cell type, the chondrocyte.

To ensure that proportions of different cell types in a tissue are maintained, constituent cells are engaged in continuous regulatory interactions via cell type-specific growth factors [12]. The self-stabilizing nature of cell interactions within a tissue also explains the fact that tissues are typically locally exclusive (i.e. one tissue in a location excludes other tissues).

The traditional distinction between tissues and organs is not necessarily categorical. Some body parts can be understood as collections of tissues, like a limb consisting of bone, connective tissue, muscles, nerves and vessels overlain by skin. Organs such as liver, lung or kidney largely consist of a highly organized ‘complex tissue’. The functionality of complex tissues depends on a spatially organized arrangement of parenchymal cells (e.g. glomeruli or loops of Henle). They also have additional scales of organization between the basic cell type, ECM complement and the whole, such as kidney nephrons or liver lobules [14]. The corresponding organ is primarily the specialized complex tissue plus ‘plumbing’ that connects it to the rest of the organism via blood vessels, nerves and ascending or descending tubes (bronchial tubes in the lung and ureter in the kidney). For this reason, we do not make a sharp distinction between ‘tissues’ and ‘organs’.

Not all metazoans have tissues as characterized above. Cnidarians and ctenophores consist of multifunctional epithelia with few mesenchymal cells. Most knowledge of tissue-level organization derives from vertebrate models. The nature of tissues in many other bilaterians is underexplored and therefore we lack a firm understanding of the phylogenetic distribution of tissue-like levels of organization. Yet we can say with confidence that metazoan body plan evolution includes the segregation of specialized cell types into separate pockets of cells with associated support, conventionally called tissues and organs. How did this occur? Two aspects of cellular metabolism are crucial for our hypothesis for the origin of supracellular organization within multicellular organisms: metabolic constraints and intercellular metabolic complementation.

### (a) Metabolic constraints

Cellular metabolism refers to chemical reactions providing free energy and biomass, which enables cells to survive, proliferate and perform their functions. Each cell can only generate a finite amount of cellular energy by utilizing a limited number of metabolic pathways such as glycolysis, the citric acid cycle, the pentose phosphate pathway and oxidative phosphorylation. Therefore, augmenting a cellular function or introducing a new function reduces the metabolic resources available for other pathways and cellular needs such as self-maintenance [15]. This is a stoichiometric constraint—a molecule of glucose can only be used once. Such trade-offs are often invoked to explain the metabolic shifts in energy and biomass production at high glucose and oxygen availability in unicellular organisms and tumour cells (Crabtree effect and Warburg effect; [16]).

Eukaryotic cells invest most of their metabolic resources in self-maintenance and performance of service functions rather than biomass increase [17]. Consider carbohydrate metabolism. It begins with the uptake of glucose from the environment and its processing to generate either: (i) energy (ATP) via glycolysis and respiration, (ii) molecular building blocks (nucleotides, amino acid (AA) precursors) or (iii) reducing molecules like NADPH to counter the oxidative stress of ATP production during respiration. Importantly, because the underlying metabolic pathways for these tasks overlap, they compete for resources and directly influence one another. This interconnectedness generates trade-offs. For example, increased ATP production via oxidative phosphorylation results in an increased reactive oxygen species (ROS) load, which in turn increases the requirement for the production of antioxidants. Similar trade-offs exist between ATP production and the synthesis of macromolecular building blocks for DNA repair, and between the replacement of damaged proteins and proliferation. Core aspects of cellular metabolism are therefore intricately interdependent: ATP production for survival and the performance of service functions, redox homeostasis enabling chemical conversions and preventing oxidative damage (to DNA, lipids and proteins), and the synthesis of building blocks for repair and proliferation. The cell is inherently limited in its ability to invest more in a specific functional role like producing and secreting enzymes or building and maintaining electrical membrane potential, because this means investing less in other aspects of metabolism.

### (b) Metabolic complementation

The phenomenon of metabolic complementation can offset the above constraints. Like unicellular organisms, cells in multicellular organisms are capable of importing and exporting a number of metabolic compounds (AAs, sugars and small metabolites like lactate, acetate), given the appropriate membrane transport mechanisms. This includes compounds that the cell is able to synthesize itself, though perhaps at an insufficient rate. Many of these compounds feed into the pathways of cellular metabolism: ATP production, redox homeostasis and building block synthesis. For example, lactate import from the extracellular space can, after oxidation to pyruvate, enter the ATP producing respiration, supplementing internal pyruvate production from glycolysis [18,19]. Furthermore, cells can exchange antioxidants and their precursors, such as cystine, the rate-limiting AA for the synthesis of the antioxidant glutathione [20]. Similarly, imported building blocks can serve in the synthesis of macromolecules.

## 3. A hypothesis for the origin of tissues and organs

**Figure 1.**
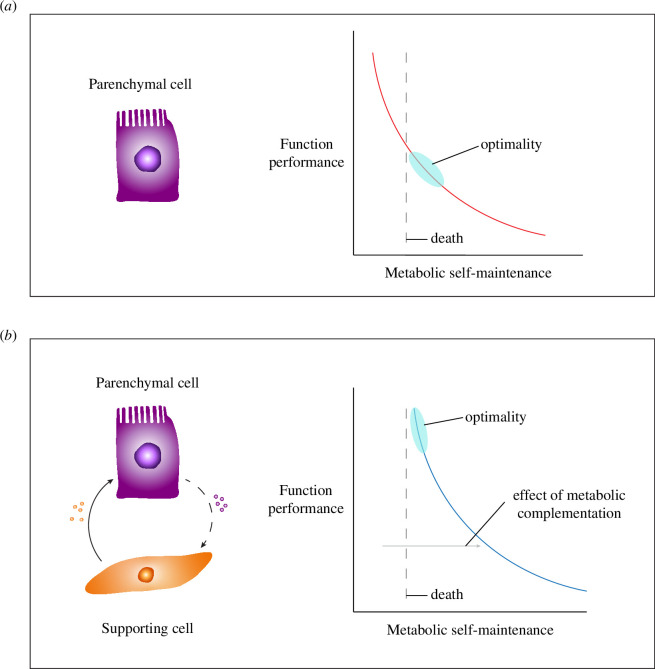
An illustration of the hypothesis of supra-functionalization. (*a*) Individual parenchymal cells are constrained by a metabolic trade-off between function performance and self-maintenance, represented by the red curve. (*b*) Metabolic complementation by supporting cells displaces the trade-off curve (blue) for the parenchymal cell further away from the limit of minimal self-maintenance required for survival. This allows the parenchymal cell to augment its functional performance in response to changes in the external or internal selective environment. As a consequence, the parenchymal cell becomes dependent on the supporting cell for survival, and the function originally performed by the parenchymal cell is now performed by a ‘tissue’.

Building on the cellular properties of constraint and complementation, we propose that the integration of pre-existing specialized cell types into higher level units consisting of different cell types is driven by the offloading of metabolic burdens from parenchymal cell types onto supporting cells (figure 1). This allows the augmentation of a specific ‘service’ function carried out by the parenchymal cell type to benefit the organism. The selective demand for such augmentation can arise due to a change in the external or internal environment. For example, a more toxic food source will favour a higher investment by the parenchymal cell into detoxification, or a new prey type (or predator) may favour more metabolic investment in locomotion. Internal selection—selection on functional relationships between traits within the organism—can have a similar effect: increases in body size and associated allometric changes in body volume may exceed the limits of diffusion processes, requiring some cell types to increase their investment into detoxifying waste products and others to specialize in the transport of resources or metabolites into and out of the body. This not only selects for the augmented service functions but also for increased investment in other functions such as transportation or skeletal support [21].

A compelling case of a metabolic trade-off between adaptive function and cell maintenance pertains to the generation of ROS. Metabolically high-performing cells require a large amount of energy. Since the oxidative processes of ATP production generate a high ROS load, this increases the cost of maintenance. Augmentation of a focal cellular function is likely to decrease the ability to invest in the cell’s maintenance, while the need for repair increases, due to the damaging effects of ROS. We propose that it is these energy and redox trade-offs that have the potential to drive the origin of tissue-level organization: augmentation of function for parenchymal cells may be enabled by the recruitment of supporting cells [22]. Empirical examples (see below) indicate that supporting cells often provide substrates for ATP generation (e.g. lactate), antioxidants (e.g. cystine) or other critical resources (e.g. glutamate for neurons), complementing the metabolism of the parenchyma and allowing increased performance of their adaptive functions.

The resulting tissue (organ) consists of specialized high-performance parenchymal cells that depend on supportive cells for their maintenance (figure 1). Thus, supporting cells contribute indirectly to the function of parenchymal cells. Once parenchymal cells rely on supporting cells for maintenance (and resources), the autonomy and performance of primary function of parenchyma is transferred to a new, supracellular unit. We propose the term ‘supra-functionalization’ for this process (supra = ‘above, beyond’) to distinguish it from familiar notions of sub- and neo-functionalization [10,23–25]. Because the parenchymal cell does not partition its function with another, similar cell, the tissue transition we describe is not a form of sub-functionalization, though this is also thought to be a mode of tissue origination [25,26]. Likewise, the transition does not involve the creation of a novel function (neo-functionalization); instead, it involves a new unit performing the same function with increased intensity: supra-functionalization.

The spatial organization of primary and supportive cells may be transient initially, responding to temporary functional needs, eventually becoming obligatory over evolutionary time [27]. Facultative spatial confinement may become permanent through the origin of a distinct ‘identity mechanism’ for a new (tissue) level of organization [14]. Identity mechanisms are expected to regulate the abundance and spatial distribution of parenchymal and supporting cell types. They become stabilized and entrenched through increased interdependencies within the tissue, such as through increased cell–cell crosstalk that supports functionality. Once interactions between the parts are entrenched, it is difficult to lose tissue-level identity without catastrophic failure of all tissue functions, giving rise to an evolutionary ‘ratchet’ mechanism—i.e. a mechanism that only permits motion in one direction [28–30].

### (a) Examples of tissue-sustaining metabolic interactions

The importance of metabolic exchange between cells for tissue redox homeostasis has long been recognized. Hermann [31] summarized observations on the brain choroid plexus and eye ciliary body, both with the function of aqueous humour production. In each case, the structures consist of humour-producing epithelium (parenchyme) and underlying stroma. It was found that epithelia show strongly positive oxidation–reduction potential, and stroma show strongly negative oxidation–reduction potential [31]. Experimental work suggested that lactate from the stroma is transported to the parenchyme, likely supplementing epithelial oxidative ATP production [31]. Similar differences between oxygen-dependent parenchyme and less sensitive non-parenchymal cells were later described in other tissues (e.g. kidney and liver; [32,33]).

Recent research on metabolic exchange between specific cells provides more detailed evidence for the strong metabolic dependency of parenchymal cells on local support cells. To illustrate this, we focus here on tissues with costly parenchymal function—brain and exocrine pancreatic tissue.

In the nervous system, glial cells play supportive roles, providing nutritional and homeostatic support for neurons. Astrocytes of the vertebrate CNS are a paradigmatic example of a support cell. Neuronal activity is costly. Astrocytes supply lactate for neurons to use in oxidation to yield ATP. Dependence of neurons on aerobic metabolism is manifest in culture: they die very quickly without oxygen [34,35]. Astrocytes store glycogen to overcome periods of low glucose, uptake glutamate from synaptic space to limit its transmitter activity and synthesize glutamine via α-ketoglutarate in the citric acid cycle, to extrude into extracellular space as a source for neuronal glutamate. Coupled to neuronal activity, astrocytes release precursors of glutathione, a major antioxidant effector, strengthening their protection against ROS [36–38]. Together with endothelial cells providing oxygen and glucose, neurons and glia form a metabolic unit [35].

Glial supportive functions for neurons pre-date their specialized roles. For example, the myelin production by the Schwann cell in the peripheral nervous system, which enhances axonal conduction speed, is a derived trait [39,40]. The phylogenetic distribution of glial abundance suggests that abundance and diversity of glial cells increased with nervous system complexity over evolutionary time: glia represent only 10–15% of cells in the *Drosophila* nervous system, 50% in mice and up to 90% in the human CNS [41]. More important than the changes in supportive cell abundance is the evolution of their metabolic contribution.

**Figure 2.**
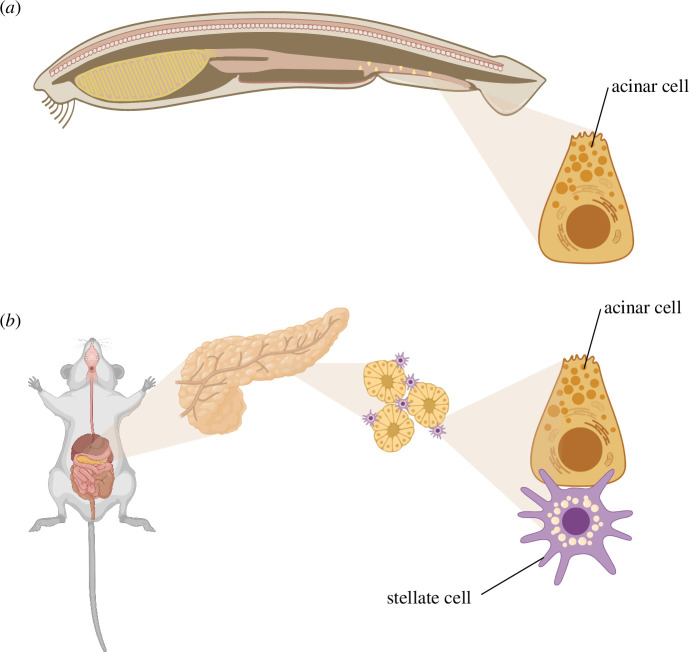
The evolution of the exocrine pancreas illustrates the proposed transition from individual cells to tissues and organs. (*a*) In early chordates, such as *Amphioxus*, ‘acinar’ cells are individually dispersed throughout the intestine. (*b*) Eventually, acinar cells were integrated into a tissue or organ structure where they are dependent on metabolic complementation from supporting stellate cells. Figure created in BioRender, with modification, J. DiFrisco (2024) BioRender.com/y04y684.

The parenchymal cells of exocrine pancreas are acinar cells, which are responsible for the production and secretion of digestive enzymes into the gut (figure 2). Enzyme synthesis and secretion are energy intensive and require high glucose and AA uptake and a high oxidative phosphorylation rate [42–45]. Correspondingly, culturing acinar cells requires special culture conditions with ECM components, high AA supplementation and high oxygenation [46]. Normal stimulation of acinar cells triggers oscillatory intracellular Ca^2+^ signalling, synchronously mediating exocytosis and mitochondrial ATP production. Sustained stimulation, in contrast, causes mitochondrial failure resulting in necrosis [47,48]. Due to a high rate of ROS production during stimulation, acinar cells rely on the cystine/glutamate antiporter system, which imports extracellular antioxidant precursor cystine in exchange for glutamate [49]. Given that the medical literature focuses on fibrosis, metabolic complementation among *healthy* pancreatic cells and the sources of acinar AA and antioxidant uptake appear to not have been studied directly. A likely candidate for metabolic complementation is the pancreatic stellate cell (PaSC), a tissue-specific fibroblast, similar to those of kidney, liver and lung. PaSCs are localized along the receptor- and transporter-rich basal pole of acinar cells. Known contributions of healthy (‘quiescent’) PaSCs to tissue homeostasis and acinar function include ECM turnover and maintenance of basal membrane [50,51] plus regulatory effects on acinar secretion [52,53]; their metabolic role is unknown. However, it is well appreciated that activated PaSCs metabolically support pancreatic ductal cancer cells [45,54–56]. It remains to be determined whether metabolic support is also delivered by quiescent PaSCs to healthy acinar cells.

The requirement for supportive cells potentially explains the origin of small composite glands in vertebrates (e.g. thyroid and parathyroid), consisting of diverse types of endocrine cells. In some vertebrates, a main gland like the thyroid hosts nests of cells of different embryonic origin and function. For the thyroid, the calcitonin-producing C-cells reside among thyroid follicles. C-cells originate from the fourth and fifth embryonal pharyngeal pouch and migrate into the thyroid in mammals, while the thyroid originates from the floor of the embryonic pharynx. In contrast, the adult teleost C-cells still reside near the posterior gill slits and not the thyroid. As metabolic requirements are generic across cells (i.e. energy, ROS and AAs), these may have aggregated to share supportive cells.

### (b) Why was metabolism overlooked in evolutionary biology?

Although well appreciated in microbial ecology and general physiology, cell metabolism and cellular metabolic exchange have not featured prominently in developmental and evolutionary biology (but see [57]). This may be due to substantial metabolic plasticity plus the ancient origin and conservation of central biochemical processes.

Ideas about cell communication have largely focused on information exchange, especially messages informing the cell about its environment to elicit a reaction (but see [31,58]). This implicitly assumes an autonomy of development and evolution from cellular metabolism. In contrast, our proposal emphasizes the exchange of metabolic compounds among animal cells. Even in the same organism, some cell types are ‘auxotrophic’ and others ‘autotrophic’ with respect to a range of AAs, even though the genome includes genes for the enzymes necessary to synthesize these. Immune cells, for example, are auxotrophic for a wide range of AAs [59]. Metabolic exchange is becoming recognized as a critical player in immune cell function [60,61]. Beyond information, therefore, cells exchange essential substances that allow, and thus can regulate, their function and survival.

### (c) Implications of the hypothesis

Our hypothesis precipitates expectations about the kind of interactions that we should observe empirically among cells in a tissue and organ. The most direct comparison that bears on why cells aggregate into tissues is between (i) organisms where the relevant primary cell types have undergone the tissue transition and become parenchyme and (ii) organisms where homologous primary cell types are not integrated into a tissue. Specifically, we expect differences in the metabolic profiles of and interactions among these cells. Parenchymal cells in tissues will (i) have more metabolic exchange with surrounding cells, (ii) be more existentially dependent on surrounding cells for self-maintenance, and thus more prone to death upon removal from the tissue and (iii) have functional augmentation compared with homologous cells outside tissues.

The most abundant tissue- or organ-wide cellular data are available at the transcriptomic level. Unlike in the study of microbial communities [62], the analysis of transcriptomic data in the study of multicellular organismal biology is seldom focused on the expression of metabolic genes (but note that the tools are becoming available [63–65]). Examples to focus on include genes for metabolic enzymes or signatures of metabolic exchange, such as genes for peptide or AA transporters. This is perhaps the most immediate source of information that can illuminate cellular metabolic homeostasis in the context of tissue organization and its evolution, apart from direct study of the metabolome.

## 4. Discussion: on the origins of within-organism levels of organization

Our hypothesis describes how an evolutionary shift of organizational level from cell type to tissue can occur without a qualitative change in functional role. Typically, the evolutionary emergence of a new level of organization is conceptualized within the ‘major transitions in individuality’ framework [66–71]. However, the tissue transition within a multicellular organism requires a different approach.

‘Evolutionary transitions in individuality’ usually refer to the emergence of new units of selection or whole-organism levels, such as the aggregation of cells into a multicellular system that can only reproduce as a whole [72–75]. A critical problem in understanding these transitions is to explain how these aggregates ensure cooperation and suppress ‘cheating’ among component cells, such as through control of proliferation and germline sequestration [66,68,71,72]. In contrast, the cell-to-tissue transition creates a new *body part* rather than a new organism level or unit of selection [24,76,77]. Instead of cooperation and conflict, the focal problem becomes understanding whether and how the tissue comes under modular genetic control, allowing it to vary and adapt quasi-independently of other body parts [14,78–80]. For example, if the supporting cells are generic to multiple tissues, this creates pleiotropic links across different traits. Such a case has been shown recently, where selection of skin fibroblasts is associated with gene expression changes in endometrial fibroblasts [81]. These links may need to be suppressed via supporting cell specialization or via tissue-specific compensation [82]. Such tissue-specific cell types belonging to the generic category of fibroblasts or macrophages are well known. Modularity at the tissue level, however, is not yet well understood.

At an abstract level, supra-functionalization involves a division of (metabolic) labour between primary and supporting cells, but it also differs from existing division of labour theories in several respects. Division of labour models picture an initially homogeneous set of components, each of which must perform the same set of adaptive tasks [83,84]. The tasks cannot all be optimized simultaneously, giving rise to trade-offs. Trade-offs can be circumvented through partitioning tasks among components (i.e. a division of labour) thereby allowing the tasks to be performed more efficiently. In our hypothesis, by contrast, primary and supportive cells already perform distinct tasks from the start. The central trade-off within primary cells is a metabolic trade-off between performance of adaptive function and self-maintenance. Crucially, in line with prevailing assumptions (§3.2), division of labour models tend to represent components as persistent ‘atomic’ units and do not consider their self-maintenance as something that can be changed, constrained and redistributed. All cells are dynamic, far-from-equilibrium systems that have to be maintained through metabolism against the physical tendency toward entropic degradation. Although this universal cellular condition gives rise to constraints on cellular adaptation, it also provides opportunities for overcoming these constraints through metabolic complementation via integration into tissues.

## Data Availability

This article has no additional data.
